# Improving palliative care with deep learning

**DOI:** 10.1186/s12911-018-0677-8

**Published:** 2018-12-12

**Authors:** Anand Avati, Kenneth Jung, Stephanie Harman, Lance Downing, Andrew Ng, Nigam H. Shah

**Affiliations:** 1 Department of Computer Science, Stanford University, Stanford, CA USA; 2 Center for Biomedical Informatics Research, Stanford University, Stanford, CA USA; 3 Department of Medicine, Stanford University School of Medicine, Stanford, CA USA

**Keywords:** Deep learning, Palliative care, Electronic health records, Interpretation

## Abstract

**Background:**

Access to palliative care is a key quality metric which most healthcare organizations strive to improve. The primary challenges to increasing palliative care access are a combination of physicians over-estimating patient prognoses, and a shortage of palliative staff in general. This, in combination with treatment inertia can result in a mismatch between patient wishes, and their actual care towards the end of life.

**Methods:**

In this work, we address this problem, with Institutional Review Board approval, using machine learning and Electronic Health Record (EHR) data of patients. We train a Deep Neural Network model on the EHR data of patients from previous years, to predict mortality of patients within the next 3-12 month period. This prediction is used as a proxy decision for identifying patients who could benefit from palliative care.

**Results:**

The EHR data of all admitted patients are evaluated every night by this algorithm, and the palliative care team is automatically notified of the list of patients with a positive prediction. In addition, we present a novel technique for decision interpretation, using which we provide explanations for the model’s predictions.

**Conclusion:**

The automatic screening and notification saves the palliative care team the burden of time consuming chart reviews of all patients, and allows them to take a proactive approach in reaching out to such patients rather then relying on referrals from the treating physicians.

## Background

The gap between the desires of patients of how they wish to spend their final days, versus how they actually spend, is well studied and documented. While approximately 80% of Americans would like to spend their final days at home if possible, only 20% do [1]. Of all the deaths that happen in the United States, up to 60% of them happen in an acute care hospital while the patient was receiving aggressive care. Over the past decade access to palliative care resources has been on the rise in the United States. In 2008, Of all hospitals with fifty or more beds, 53% of them reported having palliative care teams; which rose to 67% in 2015 [2]. However, data from the National Palliative Care registry estimates that, despite increasing access, less than half of the 7-8% of all hospital admissions that need palliative care actually receive it [3]. A major contributor for this gap is the shortage of palliative care workforce [4]. Yet, technology can still play a crucial role by efficiently identifying patients who may benefit most from palliative care, but might otherwise slip through the cracks under current care models.

We address two aspects of this problem in our study. First, physicians tend to be overoptimistic, work under extreme time pressures, and as a result may not fail to refer patients to palliative care even when they may benefit [5]. This leads to patients often failing to have their wishes carried out at their end of life [6] and overuse of aggressive care. Second, the shortage of professionals in palliative care makes it expensive and time-consuming for them to proactive identify candidate patients via manual chart review of all admissions.

Another challenge is that the criteria for deciding which patients benefit from palliative care may be impossible to state explicitly and accurately. In our approach, we use deep learning to automatically screen all patients admitted to the hospital, and identify those who are most likely to have palliative care needs. Since the criteria for identifying palliative needs could be fuzzy and hard to define precisely, the algorithm addresses a proxy problem - to predict the probability of a given patient passing away within the next 12 months - and use that probability for making recommendations to the palliative care team. This saves the palliative care team from performing manual and cumbersome chart review of every admission, and also helps counter the potential biases of treating physicians by providing an objective recommendation based on the patient’s EHR. Currently existing tools to identify such patients have limitations, and they are discussed in the next section.

## Related work

Accurate prognostic information is valuable to patients and caregivers (for setting expectations, planning for care and end of life), and to clinicians (for planning treatment) [7, 8]. Several studies have shown that clinicians generally tend to be over optimistic in their estimates of the prognoses of terminally ill patients [5, 9–11]. It has also been shown that no subset of clinicians are better at late stage prognostication than others [12, 13]. However, in practice, the most common method of predictive survival remains to be the clinician’s subjective judgment [12]. Several solutions exist that attempt to make patient prognosis more objective and automated. Many of these solutions are models that produce a score based on the patient’s clinical and biological parameters, and can be mapped to an expected survival rate.

### Prognostic tools in palliative care

The Palliative Performance Scale [14] was developed as a modification of the Karnofsky Performance Status Scale (KPS) [15] to the Palliative care setting, and is calculated based on observable factors such as: degree of ambulation, ability to do activities, ability to do self-care, food and fluid intake, and state of consciousness. The Palliative Prognostic Score (PPS) was constructed for the Palliative Care setting as well, focusing on terminally ill cancer patients [16]. The PPS is calculated with multiple regression analysis based on the following variables: Clinical Prediction of Survival (CPS), Karnofsky Performance Status (KPS), anorexia, dyspnea, total white blood count (WBC) and lymphocyte percentage. The Palliative Prognostic Index (PPI), developed around the same time as PPS, also calculates a multiple regression analysis based score using Performance Status, oral intake, edema, dyspnea at rest, and delirium. These scores are difficult to implement at scale since they involve face-to-face clinical assessment and involve prediction of survival by the clinician. Furthermore, these scores were designed to be used within the palliative care setting, where the patient is already in an advanced stage of the disease — as opposed to identifying them earlier.

### Prognostic tools in the intensive care unit

There also are prognosis scoring models that are commonly used in the Intensive Care Unit. The APACHE-II (Acute Physiology, Age, Chronic Health Evaluation) Score predicts hospital mortality risk for critically ill hospitalized adults in the ICU [17]. This model has been more recently refined with the APACHE-III Score, which uses factors such as major medical and surgical disease categories, acute physiologic abnormalities, age, preexisting functional limitations, major comorbidities, and treatment location immediately prior to ICU admission [18]. Another commonly used scoring system in the ICU is the Simplified Acute Physiological Score, or SAPS II [19], which is calculated based on the patient’s physiological and underlying disease variables. While these score are useful for the treatment team when the patient is already in the ICU, they have limited use in terms of identifying patients who are at risk of longer term mortality, while they are still capable of having a meaningful discussion of their goals and values, so that they can be set on an alternative path of care.

### Prognostic tools for early identification

There have been a number of studies and tools developed that aim to identify terminally ill patients early enough for an end-of-life plan and care to be meaningful.

CriSTAL (Criteria for Screening and Triaging to Appropriate aLternative care) was developed to identify elderly patients nearing end of life, and quantifies the risk of death in the hospital or soon after discharge [20]. CriSTAL provides a check list using eighteen predictors with the goal of identifying *the dying patient*.

CARING is a tool that was developed to identify patients who could benefit from palliative care [21]. The goal was to use six simple criteria in order to identify patients who were at risk of death within 1 year. PREDICT [22] is a screening tool also based on six prognostic indicators, which were refined from CARING. The model was derived from 976 patients.

The Intermountain Mortality Risk score is an all-causes mortality prediction based on common laboratory tests [23]. The model provides score for 30-day, 1-year and 5-year mortality risk. It was trained on a population of 71,921 and tested on 47,458.

Cowen et al. [24] proposed using a twenty-four factor based prediction rule at the time of hospital admission to identify patients with high risk of 30-day mortality, and to organize care activities using this prediction as a context. One of the their motivation was to have a rule from a single set of factors, and not be disease specific. The model was derived from 56,003 patients.

Meffert et al. [25] proposed a scoring method based on logistic regression on six factors to identify hospitalized patients in need of palliative care. In this prospective study, they asked the treating physician at the time of discharge whether the patient had palliative care needs. The trained model was then used to identify such patients at the time of admission. The model was derived from 39,849 patients.

Ramachandran et al. [26] developed a 30-day mortality prediction tool for hospitalized cancer patients. Their model used eight variables that were based on information from the first 24 h of admission, and laboratory results and vitals. A logistic regression model was developed from these eight variables and used as a scoring function. The model was derived from 3062 patients.

Amarasingham et al. [27] built a tool to screen patients who were admitted with heart failure, and identify those who are at risk of 30-day readmission or death. Their regression model uses a combination of Tabak Morality Score [28], markers of social, behavioral, and utilization activity that could be obtained electronically, ICD-9 CM codes specific to depression and anxiety, billing and administrative data. Though this study was not specifically focused on palliative care, the methodology of using EHR system data is relevant to our work. The model was derived from 1372 patients.

Makar et al. [29] used only Medicare claims data on older population (≥ 65 years) to predict mortality in six months. By limiting their model to use only administrative data, they hypothesized an easier deployment scenario thereby making automated prognostic models more prevalent. The model was derived separately on four cohorts (one per disease type) with 20,000 patients per cohort.

### Prognosis in the age of big-data

The rapid rise and proliferation of EHR systems in healthcare over the past couple of decades, combined with advances in Machine Learning techniques on high dimensional data provides a unique opportunity to make contributions in healthcare, especially in precision medicine and disease prognosis [30, 31]. All the tools described above, and those we reviewed [32–36], have at least one of the following limitations. They were either derived from small data sets (limited to specific studies or cohorts), or used too few variables (intentionally to make the model portable, or avoid overfitting), or the model was too simple to capture the complexities and subtleties of human health, or was limited to certain sub-populations (based on disease type, age etc.) We address these limitations in our work.

## Methods

We hypothesize, as described earlier, that predicting mortality is a reasonable approximation to predicting palliative needs in patients, though palliative care is applicable more broadly beyond just end of life care, including patients still undergoing painful curative treatments (such as bone marrow transplants, etc). Our approach to the problem of mortality prediction is from the point of view of the palliative care team, by being largely agnostic to disease type, disease stage, severity of admission (ICU vs non-ICU), age etc. The scale of data (in terms of number of patients) allows us to take a deep learning model that considers every patient in the EHR (with a sufficiently long history), without limiting our analysis to any specific sub-population or cohort. We frame a proxy problem statement (in place of identifying palliative needs) in a tractable way as follows:

*Given a patient and a date, predict the mortality of that patient within 12 months from that date, using EHR data of that patient from the prior year.*

This framing lends itself to be treated as a binary classification problem, and we take a supervised learning approach using deep learning to solve it. Other than building a model that performs well on the above problem, we are also separately interested in its performance on a specific patient sub-population: patients who are currently admitted. This is because it is much easier for the palliative care staff to intervene with admitted patients. This problem formulation and modeling has been previously described in [37].

### Data source

STRIDE (Stanford Translational Research Integrated Database Environment) [38] is a clinical data warehouse supporting clinical and translational research at Stanford University. The data is available in the form of a relational database, from which we extract features and creating a training dataset using SQL queries. The snapshot of STRIDE (version 6) used in our work includes the EHR data of approximately 2 million adult and pediatric patients cared for at either the Stanford Hospital or the Lucile Packard Children’s hospital between 1990 and 2014.

### Constructing a dataset for supervised learning

In constructing a supervised learning data set, we define the following concepts:

- *Positive cases*: Patients who have a recorded date of death in the EHR are considered *positive cases*.
- *Negative cases*: Patients who do not have a recorded date of death in the EHR are considered *negative cases*.
- *Prediction date*: The point in time, specific to each patient, that divides the patient’s health record timeline into virtual future and past events, is considered that patient’s *prediction date*.

Data from each patient’s virtual past is used to calculate the probability of their death 3-12 months in the future. Note that we must take care when defining the *prediction date* to not violate common sense constraints (described below) that could invalidate the labels. We only include patients for whom it is possible to find a *prediction date* that satisfies these constraints.

#### Positive cases

Palliative care is most beneficial when the referral occurs 3-12 months prior to death. The preparatory time required to start palliative care generally makes it too late for patients who pass away within three months. On the other hand, a lead time longer than 12 months is not desirable either, because making accurate predictions over such a long time horizon is difficult, and more importantly, palliative care interventions are a limited resource that are best focused on more immediate needs. The constraints that the *prediction date* should meet for positive cases are as follows:

- The *prediction date* must be a recorded date of encounter.
- The *prediction date* must be at least 3 months prior to date of death (otherwise death is too near).
- The *prediction date* can be at most 12 months prior to date of death (otherwise death is too far).
- The *prediction date* must be at least 12 months after the date of first encounter (otherwise the patient lacks sufficient history on which to base a prediction).
- In-patient admissions are preferred over other admission types for the *prediction date*, as long as they meet the previous constraints (since it is easier to start the palliative care conversation with them).
- The *prediction date* must be the earliest among the possible candidate dates subject to previous constraints.

#### Negative cases

Negative cases (patients without a date of death) are those patients who were alive for at least 12 months from the *prediction date*. Their *prediction date* is chosen such that it satisfies the following constraints:

- The *prediction date* must be a recorded date of encounter.
- The *prediction date* must be at least 12 months prior to date of last encounter (to avoid ambiguity of death after date of EHR snapshot).
- The *prediction date* must be at least 12 months after the date of first encounter (otherwise insufficient history).
- In-patient admissions are preferred over other encounter types for the *prediction date*, as long as they meet the previous constraints (to serve as controls for the admitted positive cases).
- The *prediction date* must be the latest among the possible candidate dates subject to previous constraints.

#### Admitted patients

These are patients whose *prediction date* chosen based on the above criteria happens to fall within an in-patient admission. Remaining patients (i.e, patients whose *prediction date* does not fall in range during an in-patient admission) are considered non-admitted. Note that non-admitted patients could still have other recorded admissions in their history, just their *prediction date* did not fall in one of those ranges. Further, we observe that patient records generally get updated with the latest diagnostic and physiological data, such as preliminary tests, diagnostics, etc. within the first twenty four hours of admission. Therefore the second day of admission is generally better suited for making a more informed prediction. Based on this rationale, for *admitted patients*, we re-adjust their *prediction date* by incrementing it to be the second day of admission.

**Fig. 1:**
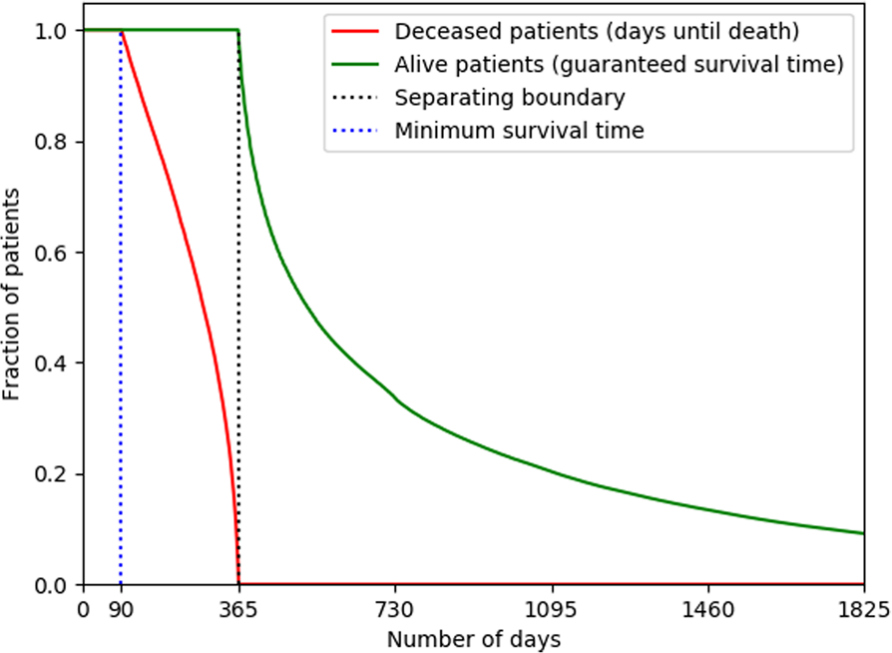
Right-censoring lengths shown as a survival plot

For both positive and negative cases, we censor all the data after their corresponding *prediction date*. The KM-plot of censor lengths is shown in Fig. 1, highlighting the separation between the two classes at 365 days.

### Data description

The inclusion criteria resulted in selecting a total of 221,284 patients. Table 1 shows the breakdown of these patients based on inclusion and admission. Note that the *admitted patients* are kept a subset of the included patients, and not separated into a disjoint set.

**Table 1:** Breakdown of patient counts

	Alive	Deceased	Total
In EHR	1,880,096	131,009	2,011,105
Selected	205,571	15,713	221,284
Admitted	9648	1131	10,779

**Fig. 2:**
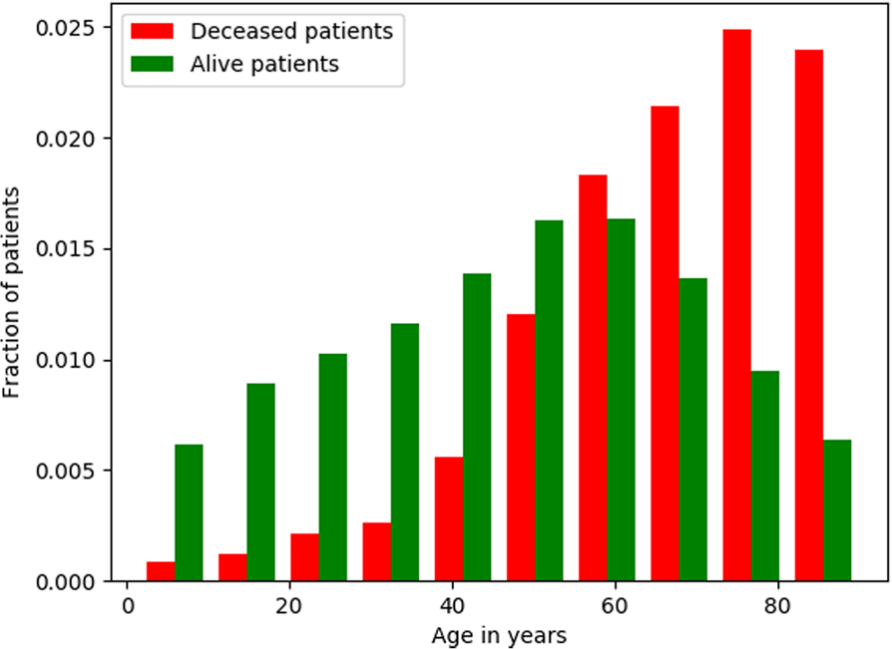
Age of patients at *prediction time*

We observe that, unsurprisingly, the distribution of age at prediction time is not equal between the classes, and that the positive class (of deceased patients) is skewed towards older age (Fig. 2).

The included patients are randomly split in approximate ratio 8:1:1 into training, validation and test sets, as shown in Table 2.

**Table 2:** Data split for modeling

	Training	Validation	Testing	
Alive	164,424	20,619	20,528	205,571
Deceased	12,587	1520	1606	15,713
	177,011	22,139	22,134	221,284

The prevalence of death among the set of all included patients is approximately 7%. Of all the included patients, approximately 5% were *admitted patients* (i.e., those who had their *prediction date* as the second day of an admission). Among the *admitted patients* subset, the prevalence of death is a little higher, at about 11%.

### Feature extraction

For each patient, we define their *observation window* as the 12 months leading up to their *prediction date*. Within the *observation window* of each patient, we create features using ICD9 (International Classification of Diseases 9th rev) diagnostic and billing codes, CPT (Current Procedural Terminology) procedure codes, RXCUI prescription codes, and encounters found in that period.

Features are created as follows. We split the *observation window* of each paitent into four *observation slices*, specified relative to the *prediction date* (PD). This is done in order to capture the longitudinal nature of the data. The exact split points of each slice within the *observation window* is shown in Table 3.

**Table 3:** Observation window and slices

	Start date	End date	Duration
*Observation window*	PD - 365	PD	365
*Observation slice 1*	PD - 30	PD	30
*Observation slice 2*	PD - 90	PD - 30	60
*Observation slice 3*	PD - 180	PD - 90	90
*Observation slice 4*	PD - 365	PD - 180	185

Thus, *observation slice 1* is the most recent, and *observation slice 4* is the oldest. In order to give more emphasis to recent data, the slice widths are intentionally narrow in the later slices compared to the earlier ones. Within each *observation slice*, we count the the number of occurrences of each code in each code category (prescription, billing, etc.) in that range. The count of every such code within the slice is considered a separate feature.

We also include the patient demographics (age, gender, race and ethnicity), and the following per-patient summary statistics in the *observation window* for each code category:

- Count of unique codes in the category.
- Count of total number of codes in the category.
- Maximum number of codes assigned in any day.
- Minimum number of codes (non-zero) assigned in any day.
- Range of number of codes assigned in a day.
- Mean of number of codes assigned in a day.
- Variance in number of codes assigned in a day.

All these features (i.e, code counts in each of the four *observation slices*, per category summary statistics over the *observation window*, and demographics) were concatenated to form the candidate feature set. From this set, we pruned away those features which occur in 100 or fewer patients. This resulted in the final set of 13,654 features. Of the 13,654 features, each patient on average has 74 non-zero values (with a standard deviation of 62), and up to a maximum of 892 values. The overall feature matrix is approximately 99.5% sparse.

### Algorithm and training

Our model is a Fully Connected Deep Neural Network (DNN) [39] having an input layer (of 13,654 dimensions), 18 hidden layers (of 512 dimensions each) and a scalar output layer. We employ the logistic function and log loss at the output layer for binary classification (with 0/1 labels), and use the Scaled Exponential Linear Unit (SeLU) activation function [40] at each layer. The model is optimized using the Adam optimizer [41], with a mini-batch size of 128 examples. The default learning rate was used (0.001).

Intermediate model snapshots of the model weights were taken every 250 mini-batch iterations, and the snapshot that performed best on the validation test was retroactively selected as the final model. Explicit regularization was not found necessary. The network configuration was reached by extensive hyperparameter search over various network depths (ranging from 2 to 32) and activation functions (*tanh*, *ReLU* and *SeLU*).

The software was implemented using the Python programming language (version 2.7), PyTorch framework [42], and the scikit-learn library (version 0.17.1) [43]. The training was performed on an NVIDIA TitanX (12 GB RAM) with CUDA version 8.0.

### Evaluation metric

Since the data is imbalanced (with 7% prevalence), accuracy can be a poor evaluation metric [44]. As an extreme case, blindly predicting the majority class without even looking at the data can result in high accuracy, though as useless such a classifier may be. The ROC curve plots the trade-off between sensitivity and specificity, and the Area Under its Curve (AUROC) is generally a more robust metric compared to accuracy in imbalanced problems, but it can also be sometimes misleading [45, 46]. In use cases where the algorithm is used to surface examples of interest based on a query from a pool of data (e.g “find me patients who are near death”) and take action on them, the tradeoff between precision and recall is more meaningful than the tradeoff between sensitivity and specificity. This is generally because the action has a cost associated with it, and precision (or PPV) informs us of how likely that cost results in utility. Therefore, we use the Average Precision (AP) score, also known as Area Under Precision-Recall Curve (AUPRC) for model selection [47].

## Results

In this section we report technical evaluation results obtained on the test set using the model selected based on the best AP score on the validation set.

**Fig. 3:**
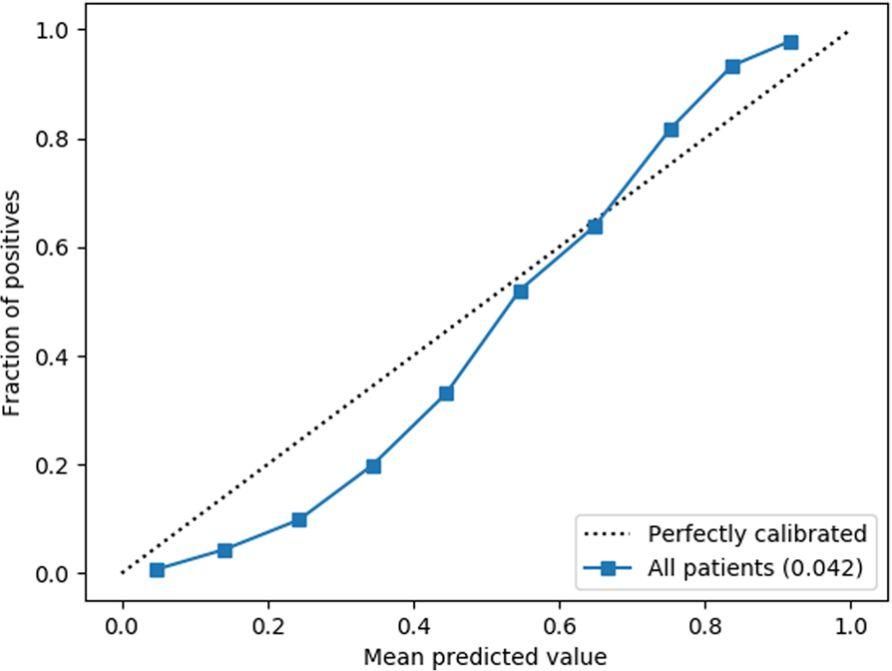
Reliability curve (calibration plot) of the model output probabilities on the test set data

We observe that the model is reasonably calibrated (Fig. 3) with a **Brier score of 0.042**. In the high threshold regime, which is of interest to us, the model is a little conservative (under-confident) in its probability estimates. This means, on average we expect the real precision in the selected candidates to be higher than expected.

**Fig. 4:**
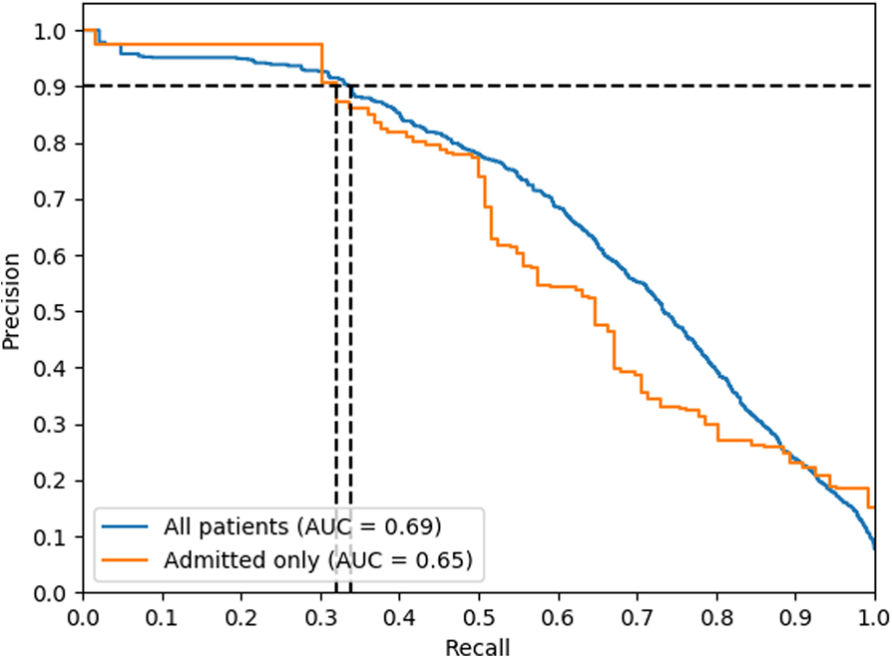
Interpolated Precision-Recall curve. The horizontal dotted line represents precision level of 0.9. The vertical dotted lines indicate the recall at which the curves achieve 0.9 precision

**Fig. 5:**
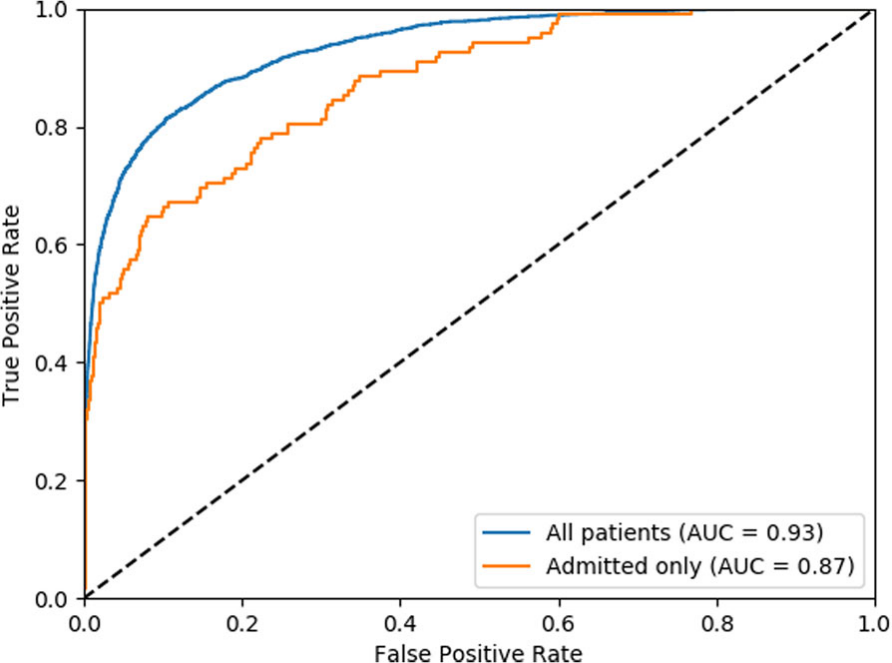
Receiver Operating Characteristic (ROC) of the model performance on the test set

The interpolated Precision-Recall curve (with the interpolation performed as explained in Chap 8 of [48]) is shown in Fig. 4. The model achieves an **AP score of 0.69** (0.65 on *admitted patients*). Early recall is desirable, and therefore Recall at precision 0.9 is a metric of interest. The model achieves **recall of 0.34 at 0.9 precision** (0.32 on *admitted patients*). The Receiver Operating Characteristic curve is shown in Fig. 5. The model achieves an **AUROC of 0.93** (0.87 for *admitted patients*). Both the ROC and Precision-Recall plots suggest that the model demonstrates strong early recall behavior.

### Qualitative analysis

It is worth recalling that predicting mortality was only a proxy problem for the original problem of identifying patients who could benefit from palliative care. In order to evaluate our performance on the original problem, we inspected false positives with high probability scores. Although such patients did not pass away within 12 months from their prediction dates, we noted that they were often diagnosed with terminal illness and/or are high utilizers of healthcare services. This can be seen in the positive and false positive examples shown in “Discussion” section.

Upon conducting a chart review of 50 randomly chosen patients in the top 0.9 precision bracket of the test set, the palliative care team found all were appropriate for a referral on their *prediction date*, even if they survived more than a year. This suggests that mortality prediction was a reasonable (and tractable) choice of a proxy problem to solve.

## Discussion

Supervised machine learning techniques, and in particular Deep Learning techniques, have recently demonstrated tremendous success in predictive ability. However, better performance often requires larger, more complex models, which comes with an inevitable cost of sacrificing interpretability. It is worth drawing a distinction between interpreting a model itself, versus interpreting the predictions from the model [49, 50]. While interpreting complex models (e.g very deep neural networks) may sometimes be infeasible, it is often the case that users only want an explanation for the prediction made by the model for a given example. The utility of such an interpretation is generally a function of the nature of action taken based on the prediction. If the action is a high-stake action, such as an automated clinical decision, it is important to establish the trust of the practitioner in the model’s decisions for them to feel comfortable with the actions, and providing explanations along with decisions help establish that trust. In the context of our work, the action is not an automated clinical decision, but rather a tool to make the workflow of a human more efficient (i.e avoid cumbersome chart reviews), and the human (i.e palliative care doctor) is always in the loop to make the decision of whether or not to initiate a consult after having a closer look at the patient’s history. In such cases, the utility of interpretations is to make the human feel that it’s worth their time to even go by the recommendations made by the model. Decision interpretation can also help identify when non-stationarity of the patient data is reaching a certain threshold. We can get an early feel for whether the patient population over time has started drifting away from the data distribution the model was trained on, when the interpretations no longer meet common sense expectations. This could suggest it may be time to either re-tune or retrain the model with more recent data.

We make the following observations to motivate our explanation technique.

- We can view the EHR data as a strictly growing log of events, and that new data is only added (nothing is modified or removed in general). This results in all our features being positive valued (as counts, means and variance of counts, etc).
- We are most interested in explaining why a model assigns high probability to a patient. We are less interested in getting an explanation for why a healthy person was given a low probability (the reasons are also much less clear: the patient did not have brain cancer, did not have pneumonia, and so on).
- Directly perturbing feature vectors (e.g sensitivity analysis or for techniques described in [49]) does not work well in our case. For example, perturbing the feature representing the ICD count for brain cancer from zero to non zero can increase the probability of death significantly, implying that it is an important factor in general. However, that is not a very useful observation for a *specific patient who does not have brain cancer*.

These observations motivate the following technique. For each ICD-9, CPT, RXCUI and Encounter type, we ablate *all occurrences* of that code from the patient’s EHR, create a new feature vector, and measure the drop in log-probability compared to the original probability. This corresponds to asking the counter-factual: all else being equal, how would the probability change if this patient was not diagnosed with XYZ, prescribed drug ABC, etc? This drop in log-probability is considered the influence the code has on the model’s decision for that patient. Demographic features are handled as follows. We zero out the age and swap the gender to the opposite sex, and measure the respective drops in probability. Finally we sort the codes in descending order by influence, and pick the top 5 in each code category. A random example of such a positive and false positive case are shown in Tables 4 and 5.

**Table 4:** Prediction explanation generated on a random positive patient with high probability score

Patient MRN	XXXXXXX			
Probability score	0.946			
Factors	Code	Value	Influence	Description
Top Diagnostic factors	V10.51	4	0.0051	Personal history of malignant neoplasm of bladder
	V10.46	5	0.0019	Personal history of malignant neoplasm of prostate
	518.5	1	0.0012	Pulmonary insufficiency following trauma and surgery
	518.82	1	0.0008	Other pulmonary insufficiency
	88.75	1	0.0006	Diagnostic ultrasound of urinary system
Top Procedural factors	88331	1	0.0017	Pathology consultation during surgery with FS
	75984	1	0.0014	Transcatheter Diagnostic Radiology Procedure
	72158	1	0.0013	MRI and CT Scans of the Spine
	Code_Type_Count	76	0.0011	Summary statistic (count of all ICD/CPT codes)
	76005	1	0.0007	Fluroscopic guidance and localization of needle or catheter tip for spine
Top Medication factors				
Top Encounter factors	Hx Scan	21	0.0012	Number of scan encounters of all types
	Inpatient	60	0.0004	Number of days patient was admitted
	Var_Codes_per_Day	8	0.0002	Summary statistic (variance in number of codes assigned per day)
	Code_Day_Count	88	0.0001	Number of days any encounter code was assigned
Top Demographic factors	Age	81	0.0010	Age of patient in years at *prediction time*

Only factors that contributed to a drop in probability score are reported

**Table 5:** Prediction explanation generated on a random false positive patient with high probability score

Patient MRN	YYYYYYY			
Probability score	0.909			
Factors	Code	Value	Influence	Description
Top Diagnostic factors	197.7	16	0.1299	Malignant neoplasm of liver, secondary
	154.1	3	0.1254	Malignant neoplasm of rectum
	287.5	1	0.0194	Thrombocytopenia, unspecified
	780.6	1	0.0171	Fever and other physiologic disturbances of temperature regulation
	733.90	1	0.0113	Other and unspecified disorders of bone and cartilage
Top Procedural factors	73560	1	0.0502	Diagnostic Radiology (Diagnostic Imaging) Procedures of the Lower Extremities
	Code_Type_Count	20	0.0491	Summary statistic (Number of unique ICD-9/CPT codes)
	74160	1	0.0381	Diagnostic Radiology (Diagnostic Imaging) Procedures of the Abdomen
	Max_Codes_per_Day	6	0.0234	Summary statistic (Maximum number of codes in any day)
	Range_Codes_per_Day	6	0.0233	Summary statistic (Range of codes across days)
Top Medication factors	283838	1	0.0619	Darbepoetin Alfa
	28889	1	0.0247	Loratadine
	Range_Codes_per_Day	5	0.0023	Summary statistic (Ranges of codes across days)
	Max_Codes_per_Day	5	0.0023	Summary statistic (Maximum number of codes in any day)
	Code_Type_Count	6	0.0015	Summary statistic (Number of unique medication codes)
Top Encounter factors	Hx Scan	19	0.2239	Number of scan encounters of all types
	Code_Day_Count	97	0.0284	Number of days any encounter code was assigned
	Outpatient	22	0.0228	Number of Outpatient encounters
	Var_Codes_per_Day	1	0.0074	Summary statistic (variance in number of codes assigned per day)
Top Demographic factors				

Only factors that contributed to a drop in probability score are reported

## Conclusion

We demonstrate that routinely collected EHR data can be used to create a system that prioritizes patients for follow up for palliative care. In our preliminary analysis we find that it is possible to create a model for all-cause mortality prediction and use that outcome as a proxy for the need of a palliative care consultation. The resulting model is currently being piloted for daily, proactive outreach to newly admitted patients. We will collect objective outcome data (such as rates of palliative care consults, and rates of goals of care documentation) resulting from the use of our model. We also demonstrate a novel method of generating explanations from complex deep learning models that helps build confidence of practitioners to act on the recommendations of the system.
